# Pre-Adult MRI of Brain Cancer and Neurological Injury: Multivariate Analyses

**DOI:** 10.3389/fped.2016.00065

**Published:** 2016-06-23

**Authors:** Jacob Levman, Emi Takahashi

**Affiliations:** ^1^Department of Medicine, Division of Newborn Medicine, Boston Children’s Hospital, Harvard Medical School, Boston, MA, USA; ^2^Athinoula A. Martinos Center for Biomedical Imaging, Massachusetts General Hospital, Harvard Medical School, Charlestown, MA, USA

**Keywords:** multivariate analysis, machine learning, fetal, neonatal, pediatric, MRI, review

## Abstract

Brain cancer and neurological injuries, such as stroke, are life-threatening conditions for which further research is needed to overcome the many challenges associated with providing optimal patient care. Multivariate analysis (MVA) is a class of pattern recognition technique involving the processing of data that contains multiple measurements per sample. MVA can be used to address a wide variety of neuroimaging challenges, including identifying variables associated with patient outcomes; understanding an injury’s etiology, development, and progression; creating diagnostic tests; assisting in treatment monitoring; and more. Compared to adults, imaging of the developing brain has attracted less attention from MVA researchers, however, remarkable MVA growth has occurred in recent years. This paper presents the results of a systematic review of the literature focusing on MVA technologies applied to brain injury and cancer in neurological fetal, neonatal, and pediatric magnetic resonance imaging (MRI). With a wide variety of MRI modalities providing physiologically meaningful biomarkers and new biomarker measurements constantly under development, MVA techniques hold enormous potential toward combining available measurements toward improving basic research and the creation of technologies that contribute to improving patient care.

## Introduction

Basic functional tasks require the coordination and cooperation of neurons distributed in multiple regions across the brain, all of which are in a state of rapid growth in pre-adult populations. Researchers focused on pre-adult populations face considerable challenges due to brain activity differences between children and adults (1–3), the structural changes that many regions undergo during development (4–7) and the recruitment of large cohorts of age-matched subjects. Important information regarding brain function is encoded in distributed patterns of brain activity and structure (8–11) and identifying these distributed patterns is particularly challenging in a pre-adult population due to a rapidly changing physiology, a high degree of brain plasticity, small brain sizes, patient motion, and an incomplete understanding of brain development.

Magnetic resonance imaging (MRI) provides a wide variety of physiological/anatomical measurements distributed across a subject’s brain, information that may assist in both clinical applications and in basic research. The most commonly used MRI method produces basic structural information related to the concentration of hydrogen protons, two of which are present in each water molecule. Since the body regulates many tissues and organs by controlling the concentration of water across membranes, structural MRI provides clinically useful tissue contrast. Perfusion MRI measures blood perfusion by tagging fast-moving hydrogen protons in the blood stream and monitoring what tissues they travel to. Functional MRI (fMRI) measures a blood oxygen level-dependent signal associated with brain activity and is an important method for monitoring brain function during an assigned task. Functional MRI is also used to monitor normal blood oxygen levels in the brain, while the subject is at rest (called resting-state fMRI). Diffusion-weighted imaging (DWI) and the closely related apparent diffusion coefficient (ADC) are focused on acquiring measurements of water diffusion that can assist in identifying a wide variety of tissue conditions. Diffusion tensor imaging (DTI) is a directional extension of DWI that measures water diffusion in many different spatial directions throughout the brain. DTI allows the tracking of tissue coherence that is typically associated with neural fiber tracts, which is extremely important for monitoring development of brain pathways. Fractional anisotropy (FA) is a scalar value that measures the strength of directionality of a DTI measurement. MR spectroscopy is often not spatially resolved but involves a single measurement that is acquired across the entire brain or at a particular region-of-interest within the brain. Spectroscopy involves investigating the effect of small radiofrequency shifts on MR signals acquired, which provides considerable information on the concentration of a variety of molecules present in the tissue.

Multivariate analysis (MVA) techniques (i.e., multivariate regression, multivariate analysis of variance, machine learning, etc.) are statistical, computational, and pattern recognition technologies that evaluate multiple variables simultaneously. MVA technologies provide a theoretical improvement over traditional univariate techniques that only examine each acquired measurement individually. MVA has particularly large potential in MRI as many physiological and anatomical parameters can be measured, new measurements are constantly under development and distributed measurements are acquired across the entire brain. The ideal way to combine a distributed set of a variety of physiological and anatomical measurements for any particular medical condition is not known *a priori*, making MVA research applied to the developing brain a fertile field of ongoing investigation. MVA techniques can be employed to discover what subregions of the brain and what physiological/anatomical measurements best help characterize different medical conditions. Furthermore, reliably assessing brain injury can assist in detection, diagnosis, and predicting a neurological condition’s development. This in turn could lead to technologies responsible for monitoring a condition’s progression as well as monitoring a subject’s response to treatment. MVA techniques can also be used to identify clinical factors and imaging parameters that are associated with important issues, such as patient outcomes.

Variations in MRI modalities allow us to acquire a wide variety of measurements distributed across the brain *in vivo*. Furthermore, brain injury and cancer can be associated with abnormal physiological/anatomical measurements of various types at a wide variety of different locations within the brain. Thus, powerful MVA techniques that combine multiple measurements have the potential to assist in identifying the physiological/anatomical conditions associated with the formation and presence of a variety of medical conditions. MVA techniques also have the potential to inform prognosis prediction.

Recent years have seen remarkable growth in interest in MVA techniques from the pediatric, neonatal, and fetal imaging research community. An excellent review article on the use of MVA classification technologies in developmental brain imaging was published in 2009 (12), however, at the time of publication the number of research studies using MVA in a pre-adult population was limited. In the years since 2009, pediatric brain MRI studies employing MVA technologies have exhibited remarkable growth warranting a thorough systematic review. This article reviews MVA techniques applied to pediatric/neonatal/fetal populations imaged with brain MRI with a focus on brain injury and cancer.

## Methods

### Multivariate Analysis Techniques

Multivariate analysis techniques can be divided into several subgroups. Multivariate statistical techniques are quite varied in their application, with a prominent example being techniques focused on the identification of measurements that are correlated with an important clinical variable. With a large set of measurements available, MVA techniques, such as multivariable linear regression (13) can be used to identify a subset of variables associated with an important clinical outcome. MVA techniques, such as multivariate analysis of variance (MANOVA) (14), can help assess the effect that changes in one variable have on dependent variables and can generally help elucidate the existing relationships between dependent and independent variables. This review will discuss many applications of multivariate statistics in a pediatric, neonatal, and fetal population, which will help to further illustrate the wide variety of potential applications of these techniques in a medical research context. Multivariate statistical techniques can also create new measurements that are a combination of existing measures, thus creating customized factors/components that can represent underlying physiological conditions, with Factor Analysis (15), Principal Components Analysis (16), and Independent Components Analysis (17) as some representative examples. These data reduction methods (summarizing many measurements with few measurements) bridge the gap between statistical analysis techniques and the closely related computational technologies that are used in an automated and semi-automated fashion.

A related class of analysis techniques have considerable overlap with multivariate statistical approaches in that many also involve measurement selection and data reduction, however, they are often considered technologies rather than statistical analysis techniques with a prime example being machine learning (18). Machine learning is divided into two main approaches: supervised and unsupervised learning. Supervised learning technologies use training data that are a collection of measurements associated with multiple groups, such as healthy controls and subjects with a known neurological condition. The training data is used to inform future predictions, allowing the computer algorithm to assign new unknown samples to one of the training groups for which it was provided example measurements. Some supervised learning algorithms include measurement selection as part of the overall technology (the process of selecting which measurements/features to rely upon for prediction), however, many do not and feature selection is often addressed as a separate topic in the scientific literature. Example supervised learning technologies include the support vector machine (19), artificial neural networks (20), linear discriminant analysis (21), random forests (22), and the generalized linear model (23), which is also referred to as a multivariate statistical analysis technique, highlighting the considerable overlap between machine learning technologies and statistical MVA techniques.

In contrast to supervised learning, unsupervised learning technologies are not provided with a set of example training data on which to base predictions from new unknown samples. Instead, unsupervised learning technologies are tasked with performing a basic level of pattern recognition on a medical imaging examination based on the analysis strategies employed by the unsupervised learning technology. This typically involves dividing a medical examination into multiple regions-of-interest that can facilitate more in-depth analyses (this process is also referred to as image segmentation). These technologies can be applied to isolating a particular structure in the brain, identifying the extent of tissue damage due to brain injury and can be used to monitor changes in a medical condition. Unsupervised learning technology can assist in the extraction of regional physiological statistics, which can play a critical role in computer-aided diagnosis systems, supporting high-level patient-wide diagnoses. Unsupervised learning technologies can also play a useful role in tissue outcome prediction research by delineating the extent of tissue damage at follow-up imaging. Unsupervised learning technologies include the ISODATA algorithm (24) and cluster analysis (25), a family of techniques that include k-means, hierarchical clustering, etc.

### Review Parameters

The search engine MEDLINE/PubMed was used for this review on May 4th, 2015. The search terms used were <Multivariate pediatric brain MRI> or <machine learning pediatric brain MRI> or <Multivariate neonatal brain MRI> or <machine learning neonatal brain MRI> or <Multivariate fetal brain MRI> or <machine learning fetal brain MRI>. This yielded 166 articles whose titles and abstracts were reviewed for appropriateness for inclusion in this review. Articles were excluded if brain MRI was not performed in a pre-adult population. Articles were excluded if they did not involve MVA that included brain MRI data acquired as an important component of the analysis (this includes measurements from MRI examinations being incorporated into the MVA, MRI informed clinical diagnoses allowing group stratification prior to MVA, etc.). Articles were excluded if they were not in English. Articles were excluded if they were focused on healthy brains, normal neurological development, and neurodevelopmental disorders, however, articles were retained for this review if they were focused on any type of brain cancer, stroke, hemorrhage, brain injury, neurological damage associated with a medical condition located outside the central nervous system and related surgeries. Articles from this search process that were not excluded were analyzed and included in this systematic review; citations within these articles deemed of interest were added as candidates for inclusion (subject to the same exclusion criteria).

## Results

The results of this systematic review are divided into subsections focusing on brain cancer, stroke, hemorrhage, cerebral palsy, brain injury, encephalopathy, non-neurological conditions that adversely affect the brain, and surgeries with adverse effects on the brain. This is followed by a section containing the remaining studies that met our review criteria but for whom very few papers were available.

### Brain Cancer

Pediatric brain cancer is characterized by uncontrolled cell growth. Multivariate analysis of pediatric brain cancer is a relatively new field of research; however, medical image analysis of brain cancer in adult populations has been the subject of considerable effort and is the subject of a recent review paper (26). Some preliminary work in pediatric brain cancer exists that did not reach the stage of evaluation of the methodologies employed (27); however, most of the studies included a self-evaluation and are summarized in Table 1. Due to the inherent heterogeneity in these datasets, it is challenging to accurately compare the results between studies. Table 1 is provided to summarize the results and facilitate comparisons and includes the study *Author, Year* of publication, *n* (the number of subjects with brain cancer included in the study), *Results* (which summarizes important findings of the approach presented) followed by a column devoted to *Noteworthy Comments*. Accuracy refers to the Overall Accuracy metric that computes the percentage of samples correctly diagnosed from a pool of both pathological and healthy subjects. The Jaccard Index is a metric that evaluates the amount of overlap between two regions-of-interest with higher values indicating greater agreement. The Area Under the Receiver Operating Characteristic Curve (AUC) is a robust metric evaluating the tradeoff between a test’s sensitivity (its accuracy on pathological subjects) and its specificity (its accuracy on healthy subjects), with higher values indicating a test that better separates the pathological and healthy groups.

**Table 1:** **Summary of multivariate analyses applied to pediatric brain tumors**.

Author	Year	*n*	Results	Noteworthy comments
Rodriguez Gutierrez et al. (28)	2013	40	Accuracy = 91–97%	Medulloblastoma, pilocytic astrocytoma, ependymoma, and posterior fossa tumor classification
Ahmed et al. (29) also Ref. (30, 31)	2011	10	Jaccard Index: 0.60	Posterior fossa tumor segmentation
Weizman et al. (32)	2012	28	Mean surface distance error: 0.73 mm	Optic pathway gliomas: segmentation, classification, and follow-up
Reynolds et al. (33)	2007	46	Error rate = 33%	MR spectroscopy
Iftekharuddin et al. (34) also Ref. (35)	2011	10	True positive = 100%	Combines T1, T2, and fluid-attenuated inversion recovery (FLAIR)
			False positive = 25%	
Tantisatirapong et al. (36)	2014	74	Accuracy = 69%	Texture analysis and feature selection
Wels et al. (37)	2008	6	Jaccard index: 0.78	Fully automatic tumor ROIs
Jansen et al. (38)	2015	316	AUC = 0.68	Survival prediction
Grech-Sollars et al. (39)	2012	61	Apparent transient coefficient in tumor (ATCT) is significantly associated with poor prognosis	Apparent transient coefficient in tumor (ATCT: change in ADC from edema to tumor core) investigated
Felicetti et al. (40)	2015	15	Meningioma is associated with development of a second neoplasm	Childhood cancer survivors treated with cranial radiation therapy were assessed with MRI and computed tomography (CT)
Sun et al. (41)	2013	33	Focal growth pattern is associated with better survival	Study focused on clinical outcomes in subjects with pediatric brainstem tumors
Youland et al. (42)	2013	351	Improved progression-free survival and overall survival associated with gross total resection informed by MRI	Study looking at prognostic factors and survival patterns in pediatric low-grade gliomas
Dorward et al. (43)	2010	40	Nodular enhancement on MRI associated with recurrence	Pilocytic astrocytomas (slow growing tumors)
Bucci et al. (44)	2004	39	Multivariate analysis: extent of resection and histologic grade were significant predictors of outcome	Investigating outcomes in pediatric gliomas
Fernandez et al. (45)	2003	80	Partial resection: worse prognosis. Optochiasmatic localization and pilomyxoid variant: worse prognosis but not independent of extent of resection	Investigating clinicopathological factors underlying prognosis in pediatric pilocytic astrocytomas
Mulhern et al. (46)	1999	18/18	Patients treated for medulloblastoma had significantly less normal white matter and lower IQ	Two groups: medulloblastoma survivors and posterior fossa tumors
Liu et al. (47)	1998	22	Radiation dose strategy (hyperfractionation) associated with better outcomes	Prognostic factors and therapeutic options in pediatric brain stem gliomas
Arle et al. (48)	1997	33	Accuracy = 58–95%	MR spectroscopy, artificial neural networks for identifying posterior fossa tumors
Shrieve et al. (49)	1992	41	Duration of symptoms >2 months prior to treatment was a significant prognostic indicator of favorable outcome	Radiation therapy for gliomas of the brainstem

**Figure 1:**
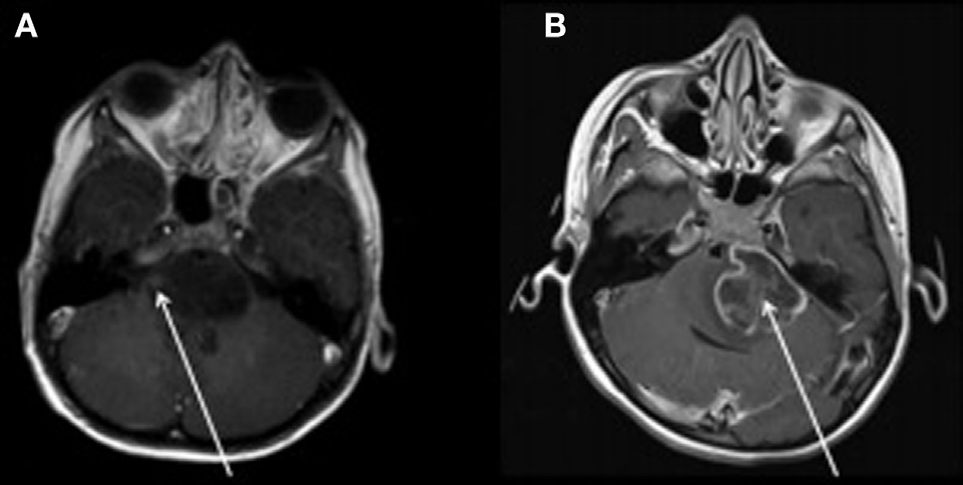
**MRI examinations of two patients with diffuse intrinsic pontine gliomas (see arrows)**. **(A)** demonstrates a small nodular enhancement (arrow) and **(B)** demonstrates a large ring enhancement (arrow). Figure reproduced with permission (38).

Magnetic resonance imaging examinations of two patients with diffuse intrinsic pontine glioma is illustrated in Figure 1 below, which is reproduced with permission from Jansen et al. (38). Diffuse intrinsic pontine glioma carries the worst prognosis of all pediatric brain tumors. Figure 1A is of a small nodular enhancement, whereas Figure 1B demonstrates a large area of ring enhancement (38).

Posterior fossa syndrome is a related disorder that occurs after posterior fossa tumor surgery in 25% of patients. The condition is characterized by mutism and disturbance of speech. Spiteri et al. (50) used multivariate techniques to determine that the presence of hypertrophic olivary degeneration is correlated with the development of posterior fossa syndrome.

### Stroke

Ischemic stroke is characterized by interrupted or severely reduced blood supply to part of the brain and is a leading cause of perinatal brain injury, cerebral palsy, and lifelong disability. Lee et al. (51) investigated maternal and infant characteristics associated with perinatal arterial stroke in infants assessed with MRI. They investigated a population of 199,176 infants identifying 40 with perinatal arterial stroke. MVA identified the following clinical characteristics as being independently associated with MRI assessed perinatal arterial stroke: history of infertility, preeclampsia, prolonged rupture of membranes, and chorioamnionitis. The rate of perinatal arterial stroke increased dramatically in the presence of multiple risk factors. Benders et al. (52) studied perinatal stroke in preterm infants using MRI and MVA and determined that preterm perinatal arterial stroke is associated with prenatal, perinatal, and postpartum risk factors. They were unable to identify any relevant maternal risk factors. Darmency-Stamboul et al. (53) investigated antenatal factors associated with perinatal arterial ischemic stroke (PAIS – a common cause of hemiplegic cerebral palsy) using MVA which indicated that maternal smoking during pregnancy was the only risk factor significantly associated with PAIS diagnosed by MRI in a section of their patient cohort. Westmacott et al. (54) investigated cognitive outcomes in children with unilateral arterial ischemic stroke and applied MVA which demonstrated that children with both cortical and subcortical lesions as assessed by MRI performed poorly cognitively relative to those with damage to either cortical or subcortical areas alone.

Sickle-cell anemia is a genetic disorder causing malformation of blood cells and reduced life expectancy and is often associated with cerebral infarctions. Dowling et al. (55) employed multivariate analyses and determined that the presence of silent cerebral infarction on MRI examinations was not associated with recurrent headaches or migraines in children with sickle-cell anemia. DeBaun et al. (56) employed multivariate regression to determine that lower baseline hemoglobin concentration, higher baseline systolic blood pressure and male gender were associated with an increased risk of silent cerebral infarction as assessed by MRI in a cohort of patients with sickle-cell anemia. Kinney et al. (57) studied subjects with sickle-cell anemia and associated silent cerebral infarcts assessed by MRI examinations in order to identify risk factors for infarction. According to the results of the MVA, patients with silent cerebral infarction were more likely to have a lower painful event rate, a history of seizures, a raised leukocyte count, and a SEN betaS globin gene haplotype.

Ischemic stroke can also be associated with hemorrhage (ruptured blood vessels) whose section of this review follows. Kirton et al. (58) combined MVA with MR findings of symptomatic neonatal arterial ischemic stroke which indicated that neonatal resuscitation was independently associated with multiple territory infarctions. MVA also indicated that bilateral lesions were an independent predictor of intracranial hemorrhage. Jordan et al. (59) investigated a pediatric population with cerebral sinovenous thrombosis and employed MVA which indicated that MRI findings, such as presence of infarction and hemorrhage, did not predict whether the subject would receive antithrombotic treatment. MVA indicated that only geographic location within the United States was a significant predictor of whether a patient would receive treatment.

### Hemorrhage

Hemorrhage is characterized by ruptured blood vessels that cause a potentially dangerous release of blood from the vasculature. Vasileiadis et al. (60) investigated uncomplicated intraventricular hemorrhage in infants at near-term age with MRI and MANOVA. Their results indicate that cerebral cortical gray matter volume was significantly reduced in the intraventricular hemorrhage group representing a 16% volumetric reduction. Their results remained statistically significant after testing for possible confounding factors and after adjusting for size differences between the infants. No differences were observed between the two groups in terms of the respective volumes of subcortical gray matter, white matter, and cerebrospinal fluid.

**Figure 2:**
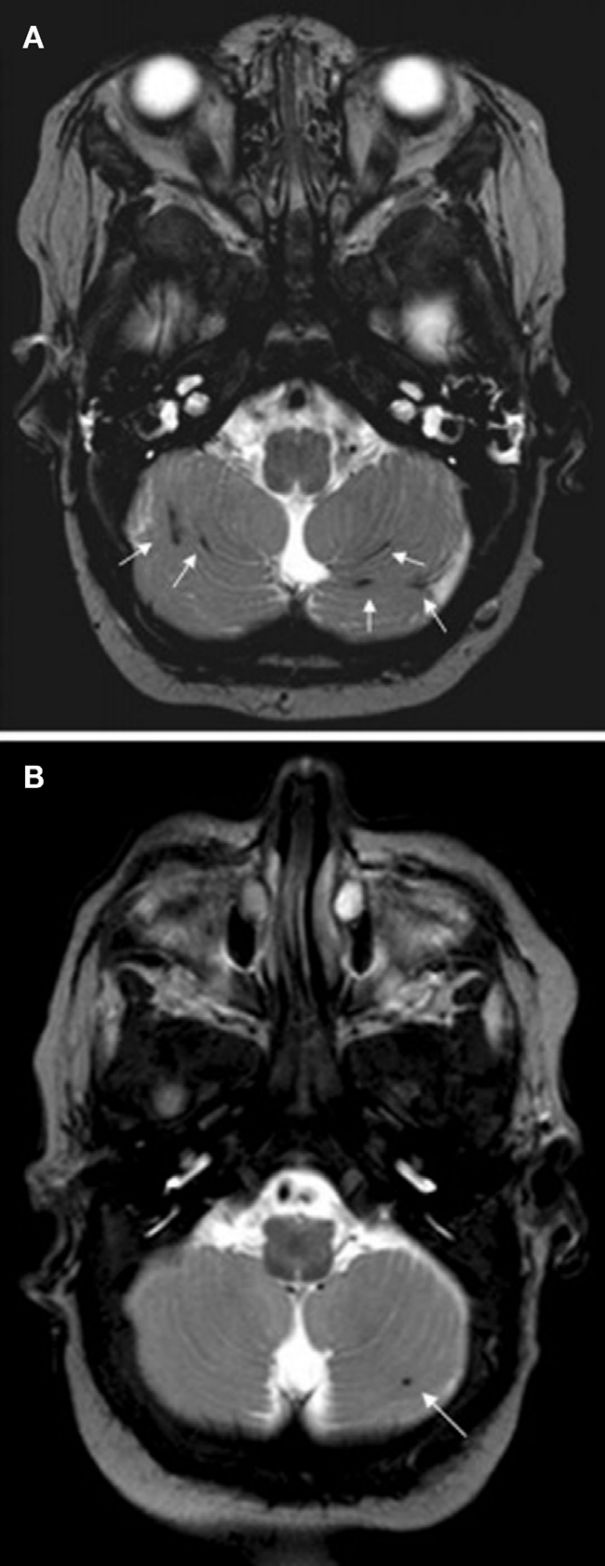
**Two preterm infants born at 29 weeks gestational age, imaged at term with T2 weighted MRI**. Subject A exhibited multiple small streak-like hemorrhages in both cerebellar hemispheres (see arrows). Subject B exhibited a single small hemorrhage in the left cerebellar hemisphere (see arrow). Figure was reproduced with permission (61).

Steggerda et al. (61) studied small cerebellar hemorrhage (CBH) in preterm infants with MRI and MVA and determined that there was no association between CBH and neurodevelopmental outcome at 2 years of age. Figure 2 illustrates two preterm infants born at 29 weeks gestational age (GA) with small CBHs (61). Wong and Fong (62) investigated children with ruptured brain arteriovenous malformations and employed multivariate analyses which demonstrated that a scoring scale that combined the Glasgow Coma Score, pupillary response and significant focal neurological injuries was a statistically significant independent predictor of clinical outcomes 6 months after hemorrhage.

Miller et al. (63) investigated the neurodevelopmental outcome of prematurely born newborns with MRI abnormalities, including hemorrhage. Their work indicated that abnormal neurodevelopmental outcome was associated with increasing severity of white matter injury, ventriculomegaly, intraventricular hemorrhage, and moderate/severe abnormalities on MRI. MVA indicated that initial severity of white matter injury as assessed by MRI and postnatal infection was independently associated with abnormal outcome.

### Trauma

Traumatic brain injury is sometimes associated with new-onset psychiatric disorders. Max et al. (64) combined DTI with multivariate analyses to determine that there was a statistically significant covariance between FA in hypothesized regions-of-interest (bilateral frontal and temporal lobes, bilateral centrum semiovale, and bilateral uncinate fasciculi) in traumatic brain injury versus orthopedic injury. Gerlach et al. (65) investigated traumatic epidural hematomas, a build-up of blood between the skull and the dura mater in children and adolescents. MVA was performed, which did not identify any variables with prognostic relevance for this population. Keenan et al. (66) performed a comparison of clinical and outcome characteristics of young children with inflicted and non-inflicted traumatic brain injury as assessed by several methods, including MRI. Their experiment included an MVA to examine the adjusted risk of a poor outcome as dependent on the type of injury. Retinal hemorrhage, metaphyseal fracture, rib fracture, and subdural hemorrhage were more common among children with inflicted traumatic brain injury relative to non-inflicted traumatic brain injury. The authors also found that 10% of inflicted traumatic brain injury would have been missed had clinicians relied only on a skeletal survey and an ophthalmologic examination. The authors recommend that such cases be referred to computed tomography and/or MRI.

Gano et al. (67) used MRI and multivariate regression to demonstrate that the odds of moderate/severe white matter injury among premature newborns decreased by 11% for each birth year of their cohort. Limperopoulos et al. (68) studied ex-preterm infants with isolated cerebellar injury using MRI and multivariate analyses and demonstrated that increases in regional cerebral volume were associated with lower odds of abnormal outcome.

Periventricular leukomalacia is a type of brain injury characterized by white matter necrosis near the lateral ventricles generally causing motor control problems or other developmental delays. Cioni et al. (69) investigated patients with periventricular leukomalacia with MRI and MVA techniques which demonstrated that visual impairment was more important than MRI lesion extent and motor disability in determining neurodevelopmental outcome in this population.

Hemiplegia is characterized by total or partial paralysis of one side of the body that results from disease or injury in the motor centers of the brain. Rocca et al. (70) investigated hemiplegia from 14 children with resting-state functional MRI, DTI, and multivariate analyses. Their results demonstrated reduced resting-state functional connectivity in the bilateral cerebellum, left precentral gyrus, and right secondary sensorimotor cortex.

### Encephalopathy

Encephalopathy is characterized by an altered mental state with common neurological symptoms, including loss of cognitive function, inability to concentrate, lethargy, and depressed consciousness. Neonatal encephalopathy is a heterogeneous group of conditions associated with lifelong developmental disabilities and neurological deficits and has a diverse etiology. Ziv et al. (71) used DTI and MVA technologies to predict neurological outcome in neonatal encephalopathy with 79% accuracy. Jenster et al. (72) used multivariate analyses to evaluate the association between maternal or neonatal infection on the severity of injury as assessed by neonatal MRI and determined that chorioamnionitis was associated with a lower risk of moderate–severe brain injury. Hayashi et al. (73) employed MVA to demonstrate that loss of consciousness 24 h after onset and prolonged seizure at onset are poor predictors of MRI assessed acute encephalopathy with reduced subcortical diffusion. Steinman et al. (74) employed MVA to a cohort of survivors of neonatal encephalopathy that demonstrated decreasing verbal intelligence quotient (IQ) with increasing basal ganglia-distribution injury as assessed by MRI.

**Figure 3:**
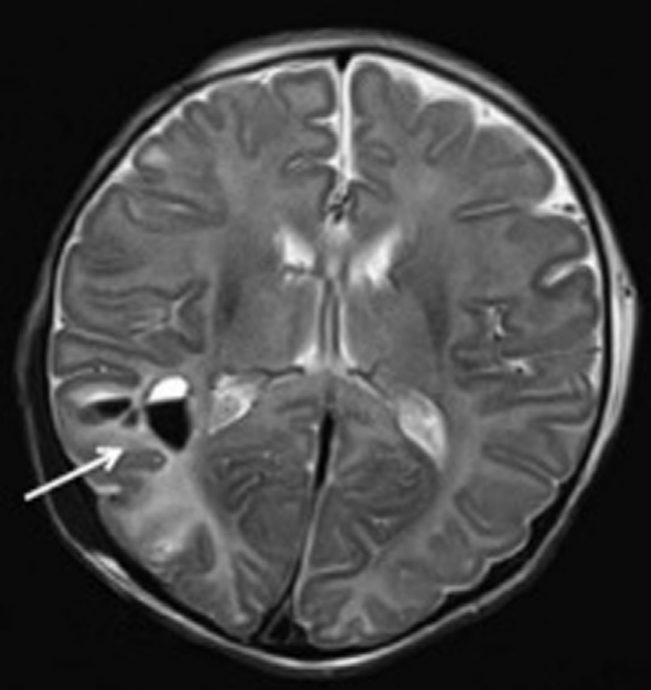
**Patterns of enhancement on T2 weighted MRI classified as severe demonstrating right intraparenchymal hematoma (arrow) with surrounding hyperintensity**. Figure is reproduced with permission (76).

Hypothermia is used as a therapeutic intervention that improves clinical outcomes and MRI findings in infants with hypoxic–ischemic encephalopathy. Wayock et al. (75) studied risk factors for severe injury in neonates treated with whole-body hypothermia for encephalopathy using MRI and multivariable logistic regression and determined that lower initial arterial pH, spontaneous respiration at >30 min of life and absence of exposure to oxytocin were associated with severe injury as assessed by MRI with 74% sensitivity and 74% specificity. Shah et al. (76) demonstrated with multivariate analyses that high seizure burden was associated with greater injury as assessed by MRI in newborns undergoing therapeutic hypothermia. Figure 3 illustrates an example right intraparenchymal hematoma with surrounding hyperintensity on a T2 weighted MRI examination of a child who received therapeutic hypothermia as a treatment for hypoxic ischemic encephalopathy. Sarkar et al. (77) used MVA to demonstrate that the existence of feeding difficulties and a history of clinical seizures were significantly associated with an abnormal MRI in patients who received hypothermia therapy. Sarkar et al. (78) demonstrated with MVA that clinically identifiable intrapartum sentinel events were independently associated with neonatal mortality related to hypoxic–ischemic encephalopathy and severe injury as shown in brain MRI, even after therapeutic hypothermia. In another study, Sarkar et al. (79) employed MVA to determine that receipt of phenobarbital before therapeutic hypothermia and placental abruption are independently associated with a worse primary outcome as assessed by neonatal death or an abnormal post-hypothermia brain MRI.

### Non-Neurological Conditions that Adversely Affect the Brain

Bronchopulmonary dysplasia is a chronic lung disorder affecting infants and children. Neubauer et al. (80) studied brain maturation in preterm infants using MRI and MVA which demonstrated that bronchopulmonary dysplasia is a predictor of delayed brain maturation. Anjari et al. (81) studied the association between lung disease and cerebral white matter abnormalities in preterm infants. The employed MVA indicated that infants receiving mechanical ventilation for more than 2 days in the perinatal period showed reduced FA in the genu of the corpus callosum and subjects with chronic lung disease exhibited a reduction in FA in the left inferior longitudinal fasciculus.

Transposition of the great arteries (TGA) is a type of heart defect characterized by a reversal of the two main arteries leaving the heart. Single ventricle (SV) physiology is another type of heart defect characterized by subjects having only a single functioning ventricle. Ibuki et al. (82) studied infants with either TGA or SV physiology with MRI and MVA to identify what anatomic development variables of the brain are associated with functional impairment. Their results demonstrated that, at 1 year old, whole and frontal lobe volumes were significantly reduced in patients with TGA and SV relative to controls. However, at 3 years old, whole and frontal brain volumes were normal in the TGA group. At 3 years old, the whole and frontal brain volumes remained significantly smaller in the SV group. Their work demonstrated that the improvement of hypoxic conditions correlates with neuroanatomic and developmental outcomes.

The placenta is an organ that connects the developing fetus to the uterine wall that allows nutrient uptake, waste elimination, and gas exchange. Reiman et al. (83) investigated whether placental inflammation is related to brain volume as assessed on MRI in preterm infants and employed MVA. Their results indicated that low GA at birth, female sex, and low birth weight correlated to smaller volumes in total brain tissue and cerebellum. Additionally, low GA and low birth weight correlated to a smaller combined volume of basal ganglia and thalami.

Diabetic ketoacidosis is a complication of diabetes that occurs when the subject’s body produces high levels of blood acids called ketones. Glaser et al. (84) studied clinical and biochemical factors influencing cerebral edema formation during diabetic ketoacidosis in children. Brain MRI was performed using the ADC and MVA identified the initial urea nitrogen concentration and respiratory rate as independently associated with an elevation in the ADC. In another study, Glaser et al. (85) investigated the frequency of sub-clinical cerebral edema (swelling) in children with diabetic ketoacidosis using MRI and MVA techniques. Swelling in turn can push on the ventricle system causing a ventricular narrowing. The results of their MVA indicated that a lower initial partial concentration of carbon dioxide (PCO_2_) level was significantly associated with ventricular narrowing. Their study concludes that ventricular narrowing is evident in just over half of children being treated for diabetic ketoacidosis and evidence of cerebral edema in affected children may be more common that had previously been reported.

Acute kidney injury is characterized by an abrupt loss of kidney function that develops within 7 days. Sarkar et al. (86) performed a MVA on a population of asphyxiated neonates with acute kidney injury. Their results indicated that abnormal MRI was more frequent in infants in the acute kidney injury group. The presence of acute kidney injury was independently associated with primary outcome.

Hypoglycemia (low blood sugar) can cause clumsiness, trouble talking, confusion, loss of consciousness, seizures, and death. Montassir (87) studied factors associated with neonatal hypoglycemic brain injury and performed a MVA which indicated that duration of hypoglycemia was statistically significantly related to hypoglycemic brain injury.

Hypogonadism is characterized by diminished functional activity of the testes and ovaries. Noetzli et al. (88) used MVA techniques based on MRI measurements of pituitary iron and volume in order to evaluate their relationship with hypogonadism. Their study found that pituitary iron and volume predict hypogonadism in patients with transfusional iron overload.

### Surgeries with Adverse Effects on the Brain

Lynch et al. (89) studied neonates with hypoplastic left heart syndrome using brain MRI and multivariate regression and determined that greater preoperative cerebral blood flow, delayed sternal closure, and a longer time between birth and surgery were predictors of postoperative white matter injury. Vinall et al. (90) studied preterm children with MRI and multivariate modeling and determined that greater numbers of invasive procedures was associated with a lower IQ and reduced white matter maturation as assessed by FA at 7 years of age. Chen et al. (91) studied stroke in infants undergoing open heart operations for congenital heart disease and their MVA indicated that lower birth weight, preoperative intubation, lower interoperative hematocrit and higher postoperative blood pressure at admission to the cardiac intensive care unit were significant factors associated with stroke as assessed by MRI. Watanabe et al. (92) studied infants with congenital heart disease and employed MVA which revealed that preoperative hypoxia is strongly associated with decreased frontal gray matter volume as well as a diagnosis of hypoplastic left heart syndrome.

Hemispherectomy is a surgical procedure involving the removal or disabling of one hemisphere of the brain. Moosa et al. (93) used multivariate analyses to demonstrate that multilobar MRI abnormalities in the contralateral hemisphere were correlated with poor language outcome in pediatric patients who had underwent hemispherectomy.

Heinrichs et al. (94) investigated adolescents after neonatal switch operation for TGA with anatomical structural MRI and MVA techniques and determined that hypoxia and preoperative acidosis were the only independent patient-related risk factors for neurological dysfunction, reduced brain volume, reduced intelligence, and periventricular leukomalacia.

### Other Studies

Cerebral palsy is a group of permanent movement disorders that develop in early childhood and is characterized by poor coordination, stiff and weak muscles, trouble swallowing or speaking, and tremors. Cerebral palsy consists of multiple subtypes, including dyskinetic cerebral palsy that is primarily associated with damage to the basal ganglia and spastic cerebral palsy that is characterized by muscles that appear stiff with unsteady movements. Griffiths et al. (95) used multivariate analyses to identify predictors that can assist in differentiating dyskinetic and spastic cerebral palsy in subjects with acute hypoxic–ischemic injury. Children with dyskinetic cerebral palsy were shown to have more frequent injury to the subthalamic nucleus. Children with spastic cerebral palsy were shown to have more severe damage to the white matter in the vicinity of the paracentral lobule.

Neurofibromatosis is a genetic condition characterized by cognitive dysfunction and carries a high risk of tumor formation, particularly in the brain. Duarte et al. (96) demonstrated that the application of MVA technologies can discriminate individuals with neurofibromatosis from controls with 94% accuracy. Huang et al. (97) created an image-matching system that retrieves similar exams to a provided case of clinical interest. Their testing of the matching algorithm included an examination of a patient with neurofibromatosis, an examination of a patient with an arachnoid cyst and a patient with an arterial venous malformation. Their image-matching results were compared to the opinion of a pediatric neuroradiologist which demonstrated that the proposed approach exhibited widely varying performance with 0–60% of the matches yielding a correct diagnosis.

Ventriculomegaly is characterized by dilation of the lateral ventricles of the brain. Baffero et al. (98) employed MVA which indicated that the only significant contribution to the prediction of major cerebral abnormalities was provided by persistence or progression of ventricular enlargement on serial ultrasound examinations. MRI findings were significant predictors.

Reiman et al. (99) studied whether genotypes that regulate individual immunologic responses are associated with MRI brain lesions and regional brain volumes in very low birth weight infants or very preterm infants. MVA was employed which indicated that the genotypes investigated were associated with reduced combined volume of the basal ganglia and thalami.

Fukuhara and Luciano (100) investigated patients with late-onset idiopathic aqueductal stenosis, a narrowing of the aqueduct of Sylvius that blocks the flow of cerebrospinal fluid in the ventricular system. Their subjects were divided into those with symptomatic headaches and those with hydrocephalus. Their study included MRI examinations and MVA which indicated that age independently affected the type of chronic symptoms. Mandell et al. (101) employed multivariate supervised learning technology to determine that large fluid volume was associated with a decrement in subject cognition.

Meningiomas are a diverse set of mostly benign tumors arising from the membranous layers that surround the central nervous system. Felicetti et al. (40) used Cox MVA in an MRI study of children who received cranial radiotherapy for cancer. Their results demonstrated that the occurrence of meningioma was associated with the development of second neoplasms and that age, sex, and cranial radiation therapy had no influence on the development of meningiomas.

Batchelor et al. (102) presented a set of measurements meant to help quantify folding patterns in the brain from fetal MRI examinations. They propose a multivariate approach to studying the shape of the cerebral cortex to assist in quantifying brain folding. Their study focused on the imaging of *ex vivo* brain specimens that included a wide variety of pathological findings, including acute chorioamnionitis, placental abruption, gastroschisis, herpes simplex viral hepatitis, placental infarcts, and fetal dyserythropoietic anemia.

**Figure 4:**
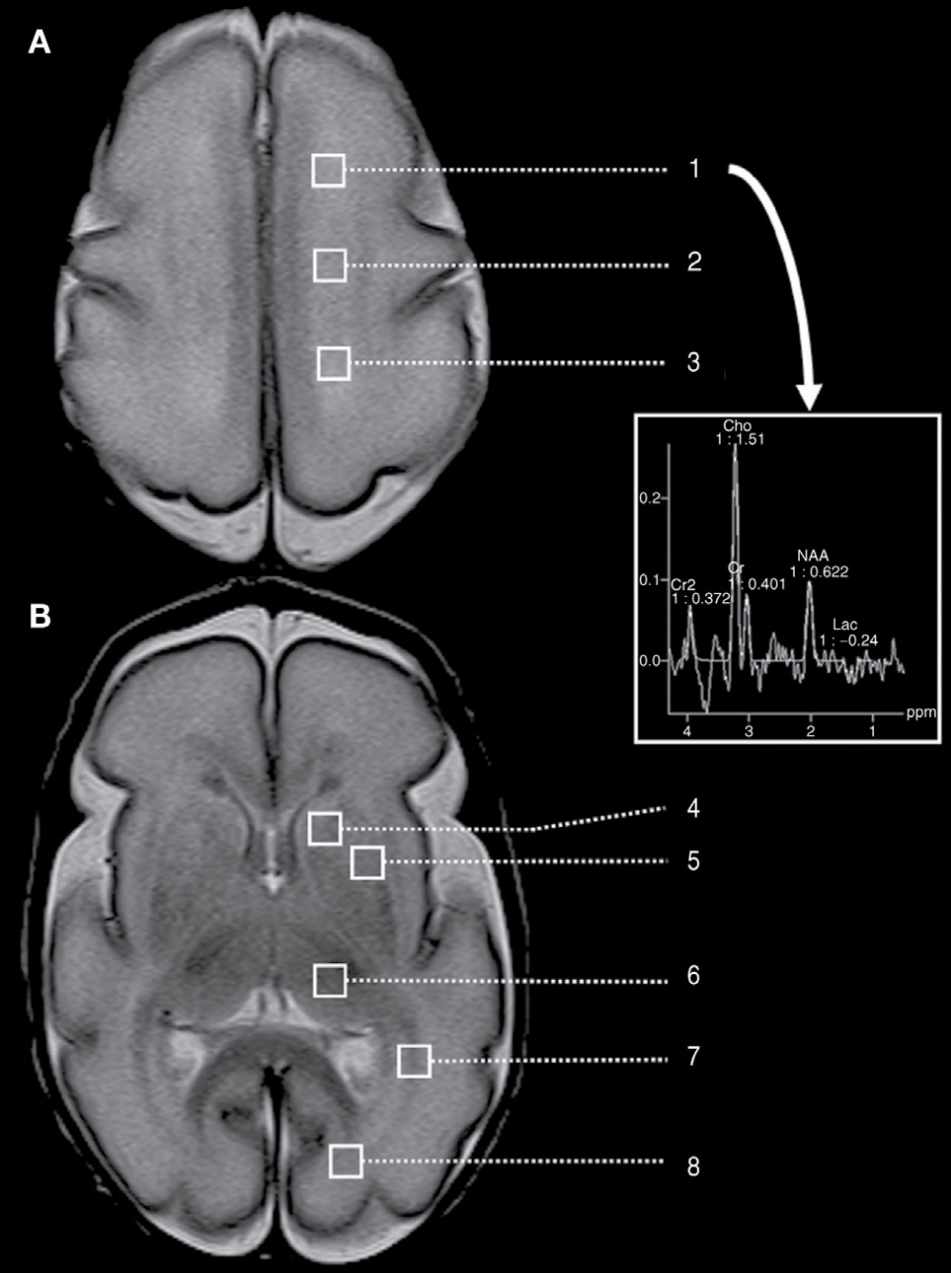
**A preterm neonate MRI examination, including spectroscopy performed at eight different of regions-of-interest (squares), located at the level of (A) the high centrum semi-ovale and (B) the basal ganglia**. This includes regions-of-interest corresponding to high white matter [(1) anterior, (2) central, and (3) posterior], (4) caudate, (5) lentiform nuclei, (6) thalamus, (7) optic radiations, and (8) calcarine region. The provided spectrum corresponds to the left frontal white matter. Cho, choline; Cr, creatine; Lac, lactate; NAA, N-acetylaspartate. Figure reproduced with permission (103).

Postnatal infection is a risk factor for adverse neurodevelopmental outcome in preterm neonates. Chau et al. (103) used multivariate regression to demonstrate that infected preterm newborns had brain imaging measures indicative of delayed brain development, such as elevated average diffusivity and decreased white matter FA. Figure 4 demonstrates a proton MRI examination, including regions-of-interest (squares) marking locations in the brain at which spectroscopy was performed. Imaging was performed on a preterm neonate born at 27 weeks GA and imaged at 28 weeks GA. Spectroscopy allows the assessment of concentration of local choline, creatine, lactate, *N*-acetylaspartate, etc., in specified regions-of-interest.

Hansen-Pupp et al. (104) investigated brain volumes and circulatory insulin-like growth factor-I in very preterm infants. Multivariate analyses indicated that higher unmyelinated white matter volume in combination female gender produced a model that was highly predictive for a Mental Development Index >85.

Bookstein et al. (105) investigated the midline corpus callosum as a neuroanatomical focus of fetal alcohol damage using MRI and MVA. Their results indicated that alcohol affected brains exhibited much more variability in the shape of the corpus callosum and its vicinity. Their work also yielded a diagnostic classification rule with 85% sensitivity and 82% specificity. Swayze et al. (106) investigated fetuses, infants, and children with fetal alcohol syndrome postmortem using MRI technology and MVA. They determined that patients with fetal alcohol syndrome have a high incidence of midline brain anomalies.

## Discussion

The results of this systematic review demonstrate a remarkably wide variety of applications of MVA techniques in brain injury and cancer among pediatric, neonatal, and fetal populations. Since the ideal combination of MVA technique and medical imaging derived clinical information is unknown *a priori* for any given application, an enormous amount of research is required to optimize MVA’s potential in this domain. Recent years have exhibited ample growth in MVA applied to brain injury and cancer with further work in this field ongoing.

While feature selection can be addressed as a class of technology separate from supervised learning, some supervised learning techniques incorporate feature selection into their learning procedure, while others do not. Feature selection is particularly important in the characterization of brain injury because we acquire a multitude of measurements distributed across the brain and we do not know *a priori* all the regions that are involved in the injury being investigated. Thus, feature selection technologies have considerable potential in improving our understanding of recovery from injury. Feature selection can theoretically lead to new technologies to assist in the characterization, detection, and diagnosis of a variety of medical conditions and to help perform clinical therapeutic assessment.

While predicting a sample as belonging to a particular group based on training data is the most common approach to the use of supervised machine learning algorithms, they can also be adapted to produce a single measurement of the severity of a given condition. This is a very powerful and underdeveloped area of research that allows the MVA technology to create composite medical images that combine multiple pre-existing images to create useful customized medical images (based on the use of the machine learning technology in regression mode, which is related to some multivariate statistical analysis techniques). Examples of customized composite medical images that can be created with this technology include images of tissue stress, images of the likelihood of the presence of injury or images of the likelihood of a given region of tissue exhibiting a physiological condition that could lead to the development of cancer.

There are many MVA techniques and technologies available to the medical researcher and there is a wide array of associated challenges that will contribute to making this an exciting and difficult research field in the years to come. One major obstacle in this application domain is caused by patient motion that is particularly challenging in children who tend to have difficulty remaining still during clinical imaging. Children instructed to remain motionless in the scanner may forget over the course of the examination. The problems with patient motion are even more poignant for many types of brain injury, such as stroke that can be disorienting for the patient and can make remaining motionless during MRI all the more challenging. Image registration is a class of technology used to compensate for patient motion but this is a very challenging problem for which no gold standard solution is available. Spatial registration to standard templates (or brain atlases) is typically based on the adult brain (107) and it has been shown that normalization procedures used can cause distortions in the brain examinations of 6-year-olds and under (108). Distortional effects may negatively impact MVA results and so care should be taken to avoid providing data containing these artifacts to MVA technology. While many studies employ image registration technology to compensate for patient motion, it should be noted that many techniques are available and those methods are not perfect and so the use of such technologies are associated with the introduction of an unknown quantity of experimental error. This unknown experimental error applies regardless of whether registration is performed for within-subject motion correction or if registration is employed to align patient data in a group-wise fashion using atlases.

Overfitting is an unwanted effect characterized by supervised learning technologies that are overly tuned to the training data provided. Overfitting can yield remarkably impressive test evaluation metrics while simultaneously not representing a robust test that will perform well on an independent dataset. Thus, reported impressive test evaluation metrics in the literature may be unrealistic if the authors are reporting results based on an overfitted MVA solution. The earliest studies targeting any given developmental condition typically have to rely on only a single dataset due to the availability of appropriate data on which to base the research. Further testing on independent datasets is required for advancing an MVA technology beyond preliminary analyses. Additionally, it is known that the number of measurements an MVA technology relies upon needs to be substantially smaller than the total number of samples provided in order to produce robust and reliable results (109). When MVA technologies are provided with an inappropriately large number of measurements (or correspondingly, too few samples), the input data space is so large that many MVA techniques might fully or nearly fully separate the data merely due to the large amount of separation between neighboring samples of any of the classes/groups provided. Thus, MVA technologies are prone to overfitting unless the number of measurements is substantially lower than the number of samples. Keeping the number of measurements at one-tenth of the total number of samples is a reasonable way to avoid this type of overfitted solution (109).

The scientific literature has seen enormous growth in developmental imaging of pre-adult populations that utilize MVA technologies. However, the majority of this work has been focused in pediatric imaging with considerably less focus on neonatal and fetal imaging. Neonatal imaging is more challenging as brain size is considerably smaller than in pediatrics and it is more challenging for imaging technicians to get a neonate to remain still over the course of their imaging examination. Patient movement induces multiple types of imaging artifacts as discussed above, which make studies on neonate populations challenging. Fetal imaging is the greatest challenge of the three as the brain sizes are the smallest and movement remains a substantial issue. Furthermore, MRI technology is reliant on the spatial proximity of a radiofrequency (RF) coil (antenna) to the organ being imaged. Normal brain imaging benefits from a specialized head RF coil that is mounted immediately adjacent to the subject’s cranium; however, in fetal imaging, this is not possible and so coils located outside the mother’s abdomen are used, inherently reducing image quality. Additional challenges exist in fetal imaging due to variations in tissue contrast observed *in utero* in fetal subjects relative to later developmental ages. Regardless of the many challenges inherent in the use of MVA in the imaging of fetal, neonatal, and pediatric populations, there is considerable potential for ongoing research growth.

## Conclusion

Multivariate analysis technologies play a useful role in helping to answer questions about structural, functional, and metabolic organization and development in the brain. Furthermore, MVA techniques have the potential to better characterize a variety of medical conditions than univariate techniques are capable of. MVA technologies have tremendous potential in the creation of the next generation of clinical diagnostic tests informed by the large amount of information acquired with clinical MRI. MVA technologies have exhibited enormous growth in MRI of pre-adult populations with a strong emphasis on pediatric imaging. The technologies are very flexible and a wide range of potential applications have already been investigated; however, so many variations on MVA technologies are available in the scientific literature that ample research will need to be performed in order to thoroughly evaluate the tradeoffs imposed by the selection of a given MVA technique applied to a particular medical condition. Future work will look at improving MVA techniques and adapting them to better characterize, diagnose, and detect neurological developmental disorders in pre-adult populations as well as to assist in the identification of clinical variables associated with important aspects of patient outcome.

## Author Contributions

Both authors were involved in the planning and design of the study. JL performed the literature review and authored the first draft of the paper. Both authors participated in editing the manuscript and approving it for submission.

## Conflict of Interest Statement

The authors declare that the research was conducted in the absence of any commercial or financial relationships that could be construed as a potential conflict of interest. The reviewers and handling Editor declared their shared affiliation, and the handling Editor states that the process nevertheless met the standards of a fair and objective review.
